# A 3D-printed molybdenum-containing scaffold exerts dual pro-osteogenic and anti-osteoclastogenic effects to facilitate alveolar bone repair

**DOI:** 10.1038/s41368-022-00195-z

**Published:** 2022-09-05

**Authors:** Beimin Tian, Xuan Li, Jiujiu Zhang, Meng Zhang, Dian Gan, Daokun Deng, Lijuan Sun, Xiaotao He, Chengtie Wu, Faming Chen

**Affiliations:** 1 Department of Periodontology, State Key Laboratory of Military Stomatology and National Clinical Research Center for Oral Diseases, Shaanxi Engineering Research Center for Dental Materials and Advanced Manufacture, School of Stomatology, Fourth Military Medical University, Xi’an, China; 2 State Key Laboratory of High-Performance Ceramics and Superfine Microstructure, Shanghai Institute of Ceramics, Chinese Academy of Sciences, Shanghai, China

**Keywords:** Regeneration, Periodontitis, Biomedical engineering

## Abstract

The positive regulation of bone-forming osteoblast activity and the negative feedback regulation of osteoclastic activity are equally important in strategies to achieve successful alveolar bone regeneration. Here, a molybdenum (Mo)-containing bioactive glass ceramic scaffold with solid-strut-packed structures (Mo-scaffold) was printed, and its ability to regulate pro-osteogenic and anti-osteoclastogenic cellular responses was evaluated in vitro and in vivo. We found that extracts derived from Mo-scaffold (Mo-extracts) strongly stimulated osteogenic differentiation of bone marrow mesenchymal stem cells and inhibited differentiation of osteoclast progenitors. The identified comodulatory effect was further demonstrated to arise from Mo ions in the Mo-extract, wherein Mo ions suppressed osteoclastic differentiation by scavenging reactive oxygen species (ROS) and inhibiting mitochondrial biogenesis in osteoclasts. Consistent with the in vitro findings, the Mo-scaffold was found to significantly promote osteoblast-mediated bone formation and inhibit osteoclast-mediated bone resorption throughout the bone healing process, leading to enhanced bone regeneration. In combination with our previous finding that Mo ions participate in material-mediated immunomodulation, this study offers the new insight that Mo ions facilitate bone repair by comodulating the balance between bone formation and resorption. Our findings suggest that Mo ions are multifunctional cellular modulators that can potentially be used in biomaterial design and bone tissue engineering.

## Introduction

An increasing number of patients worldwide suffer from degenerative bone diseases or large defects caused by injuries and tumors, resulting in severe health problems, such as long-term pain and physical disabilities. Although human bone has an inherent regenerative capacity, clinical therapies are hindered by the limited potential of current bone substitute materials to stimulate the necessary cellular response to form sufficient new bone, particularly in large bone defects.^1^ The development of three-dimensional (3D) bone scaffolds with bone-mimicking microstructure and composition together with the incorporation of proper cell modulators into the 3D compartment of the scaffolds has the potential to improve the bone regeneration outcomes of clinical procedures.^2^

As one of the most active organs, alveolar bone undergoes constant remodeling under physiological conditions.^3^ The opposing but coordinated activity of bone-forming osteoblasts and bone-resorbing osteoclasts is required to maintain the balance between bone formation and bone resorption. In diseases such as periodontitis, however, this balance is disrupted, and both osteogenic and osteoclastogenic processes are significantly altered.^4–6^ During the development of periodontitis, inflammation-related immune cells, such as proinflammatory macrophages and T helper (Th) cells, among others, can directly promote osteoclastogenesis by highly expressing receptor activator for nuclear factor-κB ligand (RANKL)^7,8^ and simultaneously inhibit osteogenesis by secreting proinflammatory cytokines, such as tumor necrosis factor (TNF)-α and interferon (IFN)-γ.^9–11^ Therefore, the positive regulation of osteogenesis plus the negative feedback regulation of osteoclastogenesis are of equal importance when developing therapeutic strategies for alveolar bone regeneration. However, most biomaterials for bone tissue engineering are designed with a focus on their capacity to improve osteogenesis; the role of anti-osteoclastic processes in supporting bone regrowth has unfortunately been largely ignored in many if not all current biomedical engineering designs.^12,13^

In support of the idea that osteogenesis is required for successful bone regeneration, the delivery of growth factors to implants and the optimization of material properties (e.g., stiffness or pore size), either alone or in combination, have been demonstrated to be reliable approaches.^14–16^ In contrast, cytokines or drugs such as osteoprotegerin, raloxifene, estrogen, bisphosphonates, calcitonin, and parathyroid hormone (PTH) are all good choices for endowing biomaterials with anti-osteoclastic functions.^15^ However, the regulatory functionality of an implant tends to decrease over time in vivo due to the suboptimal biodistribution of the released bioactive reagents and the reduction of their bioactivity due to enzymatic degradation.^17^ In contrast to frequently used therapeutic agents such as proteins, biomolecules and drugs, bioactive elements (*e.g*., silicon, strontium, manganese, magnesium and lanthanum) delivered by and released from transplants during degradation of the material can be resistant to enzymolysis; these bioactive ions have been found to exert long-term multifunctional effects to promote tissue regeneration.^18,19^ In particular, many bioactive ions have been demonstrated to promote osteogenesis while suppressing osteoclastogenesis in a number of bone disease models, suggesting that bioactive ions may play more roles in the design of bioactive materials for regenerative purposes.^9,20–22^

Among bioactive ions used as cell modulators, molybdenum (Mo) is an essential trace element for nearly all organisms and forms the catalytic center of more than fifty enzymes, such as nitrogenase, nitrate reductases, sulfite oxidase and xanthine oxidoreductase.^23^ Accumulating evidence has shown that these Mo-containing enzymes play important roles in maintaining metabolic homeostasis by catalyzing important redox reactions.^24^ In humans, Mo deficiency can lead to severe developmental disorders, which often cause death during early childhood.^24,25^ In the field of biomaterials, the incorporation of Mo elements into dental implants or bone scaffolds has been shown to significantly improve their biocompatibility and improve their ultimate reparative outcome.^26–28^ Previously, we found that Mo ions released from scaffolds exhibited robust immunomodulatory activity by targeting mitochondrial function and immunometabolism of macrophages during the wound healing cascade, leading to the coordinated regeneration of hybrid tissues.^29^ Although that work took an important step toward uncovering the role of Mo ions in tissue regeneration from the standpoint of immunomodulation by bioactive materials, and previous studies have demonstrated the pro-osteogenic capacity of Mo ions,^28^ the contribution of Mo ions to anti-osteoclastogenic cellular responses and, particularly, to the orchestration of the balance between osteoblasts and osteoclasts across the bone healing cascade remains unexplored.

Building on our previous work, in the present study, a Mo-containing bioactive glass ceramic scaffold (BG-scaffold) with solid-strut-packed structures (Mo-scaffold) was printed, and its ability to regulate pro-osteogenic and anti-osteoclastogenic cellular responses was evaluated in vitro and in vivo. First, the cellular effects of Mo-scaffold powder extracted on the osteogenic differentiation of bone marrow mesenchymal stem cells (BMMSCs) and differentiation of osteoclast progenitors were examined using in vitro cell incubation systems. Given that mitochondria are important target organelles for Mo ions^29^ and that mitochondria-rich osteoclasts are also derived from monocyte/macrophage lineages in a state of high-energy demand to facilitate bone resorption,^30,31^ the activity of mitochondrial biogenesis and production of reactive oxygen species (ROS) were evaluated to interrogate the primary mechanism behind the Mo ion-induced decrease in osteoclast differentiation. Finally, the bone regeneration outcome following in vivo Mo-scaffold transplantation and the involved synergistic cellular responses of osteoclasts and osteoblasts were evaluated in a canine critical-size alveolar bone defect model. Combined with our previous findings, these new insights into the dual therapeutic effects of Mo ions on pro-osteogenic and anti-osteoclastogenic processes will ultimately provide a new perspective for the design of novel multifunctional materials in bone regenerative medicine.

## Results

### Characterization of 3D-printed scaffolds

**Fig. 1:**
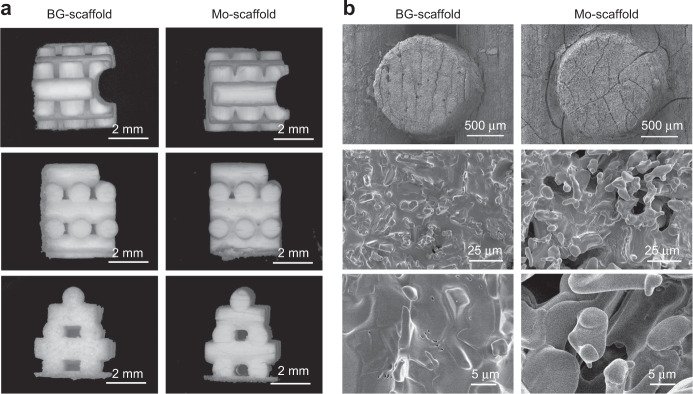
Characterization of the 3D-printed bioactive glass ceramic scaffolds with or without molybdenum (Mo-scaffolds and BG-scaffolds). **a** Representative optical microscopy images showing the gross appearance of the bone defect-fitted BG- and Mo-scaffolds (top panel: top view showing the grooves specially designed to match the tooth roots when they are placed into the bone defects; middle and bottom panels: side and bottom views showing the solid-strut-packed material structures designed for cell adhesion). Scale bar = 2 mm. **b** Representative SEM images showing the similar surface morphology of BG- and Mo-scaffolds (top panel, low magnification, scale bar = 500 µm; middle and bottom panels, high magnification, scale bars = 25 µm and 5 µm, respectively)

The precisely customized BG- and Mo-scaffolds for alveolar bone defect placement were successfully fabricated via DLP-based 3D printing. The overall shape, surface microstructure and inner compartments of the BG- and Mo-scaffolds were characterized by optical microscopy and scanning electron microscopy (SEM). As shown by optical microscopy, both BG- and Mo-scaffolds were packed with struts (diameter, 1 mm), and a groove was specially designed on one side of the scaffold to fit the tooth roots; the compartments between struts could provide sufficient space for tissue ingrowth (Fig. 1a). SEM images showed that both types of scaffolds displayed similar surface microstructures, but visible and irregular micropores were more frequently observed on the strut walls of the BG-scaffold (Fig. 1b). When the elemental distribution of Mo, oxygen (O), silicon (Si), calcium (Ca) and phosphorus (P) was visualized by energy-dispersive X-ray spectrometry (EDS) analysis, O, Si, P, and Ca were present in both scaffolds, but only Mo-scaffolds contained Mo; the atomic percentages of O, Si, P, and Ca in the BG- and Mo-scaffolds are shown in Fig. S1a. Next, the degradation behavior and ion release profiles of BG- and Mo-scaffolds were evaluated by immersing the BG- and Mo-scaffolds in Tris-HCl solution. The concentrations of Mo, Si, P and Ca in the Tris-HCl solution at each time point (3, 7, 10, 14, 21, and 28 days) were measured using inductively coupled plasma–mass spectrometry (ICP–MS). Compared to BG-scaffolds, the Mo-scaffolds showed significantly lower Si, Ca and P release. In contrast, the concentration of Mo ions released from the Mo-scaffolds was significantly higher than that released from the BG-scaffolds (Fig. S1b). Furthermore, the incorporation of Mo ions into the scaffold reduced the material degradation rate as the weight loss of the BG-scaffold was significantly larger than that of the Mo-scaffold (Fig. S1c). Finally, the concentrations of Mo, Si, Ca and P in the filtered BG-scaffold extracts (BG-extracts) and Mo-scaffold extracts (Mo-extracts) were analyzed via ICP-MS. Consistently, almost no Mo ions could be detected in the BG-extracts, while the concentration of Si ions was significantly higher in BG-extracts than in Mo-extracts. When the dilution ratio was lower than 1/32, there were no differences in the concentrations of Ca and P between BG- and Mo-extracts (Table S2).

### Mo-extract promoted osteogenic differentiation of BMMSCs

**Fig. 2:**
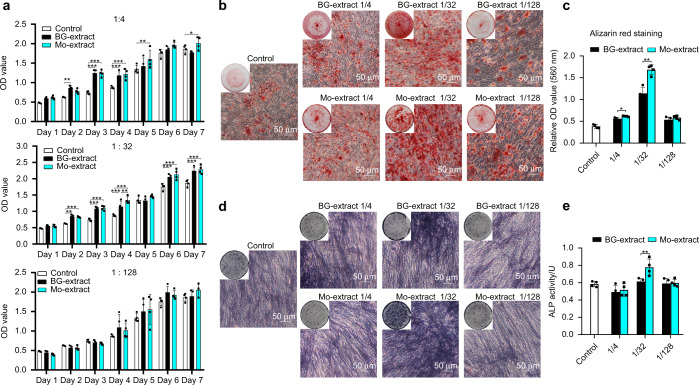
Effects of graded concentrations of diluted solutions (1/4, 1/32, or 1/128: extract solution/total medium volume) of bioactive glass ceramic powder extract with or without molybdenum (Mo-extract or BG-extract, respectively) on the osteogenic differentiation potential of BMMSCs; culture medium was used as the negative control (Control). **a** Proliferation capacity of BMMSCs incubated in graded concentrations of diluted BG- or Mo-extract solutions (*n* = 4). **b** Representative images of mineralized nodules formed by BMMSCs following a 7-day osteogenic incubation in graded concentrations of diluted BG- or Mo-extract solutions (Alizarin Red S staining; scale bar = 50 μm). **c** Quantitative analysis of the mineralized nodules formed by BMMSCs following a 7-day osteogenic incubation in graded concentrations of diluted BG- or Mo-extract solutions (*n* = 4). **d** Representative alkaline phosphatase (ALP) staining images following a 7-day osteogenic incubation of BMMSCs in graded concentrations of diluted BG- or Mo-extract solutions. Scale bar = 50 μm. **e** Quantitative analysis of ALP activity of BMMSCs following a 7-day osteogenic incubation in graded concentrations of BG- or Mo-extracts (*n* = 4). The data are shown as the mean ± SD; *P < 0.05 and **P < 0.01 indicate significant differences between the indicated columns

BG-extract or Mo-extract was diluted with culture medium at a ratio of 1/4, 1/32, or 1/128 (extract solution/total medium volume) to represent high, medium and low extract dilute solutions. The proliferation capacity of BMMSCs incubated in diluted solutions of BG-/Mo-extract was analyzed using CCK8 assays. BMMSCs incubated in diluted solutions of either BG- or Mo-extract exhibited a trend toward increased proliferation, irrespective of the dilution ratio (1/4, 1/32, or 1/128). In this context, only cells incubated in either powder extract with a dilution ratio of 1/32 displayed a significant increase in proliferation compared to cells incubated in culture medium; the Mo-extracts exhibited more significant proliferation-enhancing potential than BG-extracts (Fig. 2a). When the effects of graded diluted solutions of BG-extract or Mo-extract on osteogenic differentiation were evaluated using Alizarin Red S staining and ALP staining assays (Fig. 2b, e), BMMSCs incubated in BG- or Mo-extracts with a dilution ratio of 1/4, 1/32, or 1/128 exhibited robust potential to undergo osteogenic differentiation and form Alizarin Red S-positive mineral nodules and ALP-positive cells, and cells incubated in a 1/32 dilution ratio of Mo-extract exhibited more calcium deposition and higher cellular ALP activity (Fig. 2b, d). Quantitative analysis of Alizarin Red S staining further revealed that the Mo-extract at a dilution ratio of 1/4 or 1/32 led to greater mineralized nodule formation in BMMSCs than the BG-extract at the same dilution ratio (Fig. 2c). Consistently, the Mo-extract at a 1/32 dilution ratio exhibited more significant potential to increase cell ALP activity than the diluted BG-extract solution (Fig. 2e).

### Mo ions in culture medium exhibited potential similar to that of Mo-extract for enhancing cellular osteogenic differentiation

**Fig. 3:**
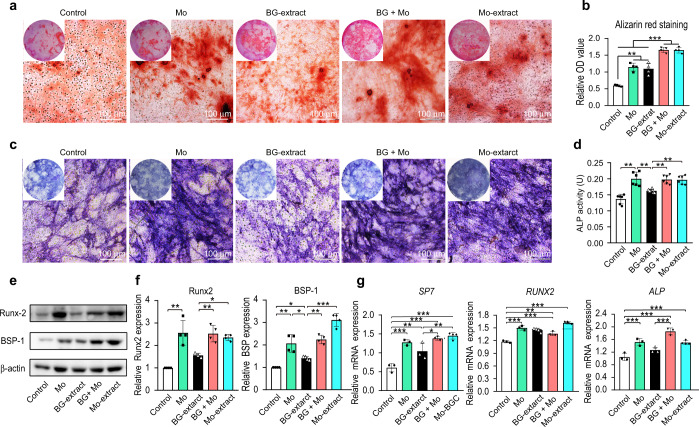
Effects of the presence of Mo ions in culture medium on the osteogenic differentiation potential of BMMSCs. Culture medium containing Mo ions (Mo) and diluted solutions (dilution ratio: 1/32) of BG-extracts containing Mo ions (BG + Mo) were set as the experimental groups, while culture medium (Control), diluted (dilution ratio: 1/32) BG-extract solution, and diluted (dilution ratio: 1/32) Mo-extract solution were set as the controls. **a** Representative images of mineralized nodules formed by BMMSCs following a 7-day osteogenic incubation in different solutions (Alizarin Red S staining; scale bar = 100 μm). **b** Quantitative analysis of mineralized nodules formed by BMMSCs following a 7-day osteogenic incubation in different solutions (*n* = 4). **c** Representative alkaline phosphatase (ALP) staining images following a 7-day osteogenic incubation of BMMSCs in different solutions. Scale bar = 100 μm. **d** Quantitative analysis of ALP activity following a 7-day osteogenic incubation of BMMSCs in different solutions (*n* = 4). **e** Expression of osteogenic proteins (Runx-2 and BSP-1) in BMMSCs following a 7-day osteogenic incubation in different solutions (Western blotting). **f** Quantitative analysis of osteogenesis-related proteins in cells following a 7-day osteogenic incubation in different solutions (*n* = 3). **g** Relative mRNA expression levels (normalized to β-actin) of osteogenesis-related genes (*SP7*, *Runx2*, and *ALP*) in BMMSCs following a 7-day osteogenic incubation in different solutions (qRT–PCR assay, *n* = 3). The data are shown as the mean ± SD; *P < 0.05, **P < 0.01, and **P < 0.01 indicate significant differences between the indicated columns

Given that the Mo-extract at a dilution ratio of 1/32 exhibited a significantly positive influence on cellular osteogenic differentiation, we further investigated how the presence of Mo ions at the same concentration present in the Mo-extract at a dilution ratio of 1/32 (3.75 mg·L^−1^) in culture medium (culture medium or 1/32 diluted solution of BG-extract) affected the osteogenic potential of BMMSCs, with the purpose of interrogating the role of Mo ions in the enhanced pro-osteogenic effects of Mo-extract compared to BG-extract. As demonstrated by Alizarin Red S staining and ALP staining, the presence of Mo ions in either culture medium or diluted solutions of BG-extracts significantly increased the formation of calcium deposits and ALP positivity after a 7-day induction (Fig. 3a–d). Quantitative analysis of Alizarin Red S staining and ALP activity indicated that the formation of mineralized nodules and ALP activity in cells incubated in diluted solutions of BG-extracts containing Mo ions were similar to those of cells incubated in diluted solutions of Mo-extracts (Fig. 3b, d). Furthermore, both Western blot and qRT–PCR analyses indicated that the presence of Mo ions in either culture medium or diluted solutions of BG-extracts significantly increased the expression of osteogenesis-related proteins (Runx2 and BSP-1) (Fig. 3e, f) and genes (*SP7* and *ALP)* in BMMSCs (Fig. 3g). In addition, the gene expression levels of *Runx2* in cells incubated in culture medium containing Mo ions were significantly higher than those in cells incubated in culture medium (Fig. 3g).

### Mo-scaffolds supported the growth of osteoclast progenitors

**Fig. 4:**
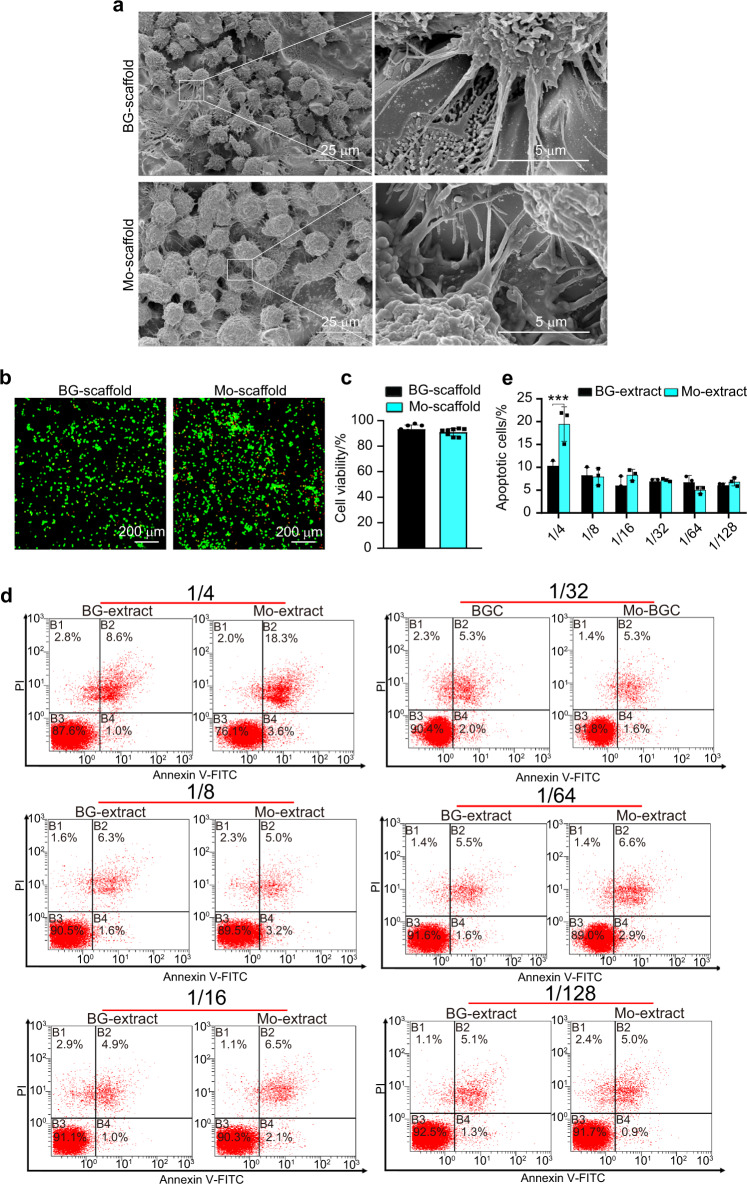
In vitro cytocompatibility test of osteoclast progenitors cultured in 3D-printed BG-and Mo-scaffolds or incubated in graded concentrations of diluted BG- and Mo-extract solutions. **a** Representative SEM images showing robust osteoclast progenitors residing on the surfaces of both scaffolds (left panels: low magnification, scale bar = 25 µm; right panels: high magnification, scale bar = 5 µm). **b** Fluorescence staining of osteoclast progenitors (live/dead) grown on BG- and Mo-scaffolds; viable cells were stained with calcein-AM (green fluorescence), while dead cells were stained with PI (red fluorescence). Scale bar = 200 µm. **c** Quantitative analysis of cell viability when osteoclast progenitors were grown on BG- or Mo-scaffolds based on calcein-AM/PI staining (*n* = 8). **d** Representative flow cytometry images showing the apoptotic cells when osteoclast progenitors were incubated in graded concentrations of diluted BG- or Mo-extract solutions (1/4, 1/8, 1/16, 1/32, 1/64, or 1/128: extract solution/total medium volume) for 7 days. **e** Quantitative analysis of apoptotic cells based on flow cytometry analysis (*n* = 3). The data are shown as the mean ± SD (*n* = 3); ***P < 0.001 indicates significant differences between the indicated columns

Next, the cellular responses of osteoclast progenitors to the BG- and Mo-scaffolds were investigated. After seeding osteoclast progenitors into BG- or Mo-scaffold for 24 h, SEM observation showed that both scaffolds supported the attachment of osteoclast progenitors, and cells closely adhered to the scaffolds via extensive sheet-like lamellipodia and filopodia extending in different directions (Fig. 4a). Live/dead cell staining indicated that most of the cells that attached to the BG- and Mo-scaffolds were viable (labeled with green fluorescence; Fig. 4b). Quantitative analysis of cell viability showed that the ratio of live osteoclast progenitors grown on either the BG- or Mo-scaffolds was more than 90%, and no significant difference in cell viability was observed between the BG- and Mo-scaffold groups (Fig. 4c). In terms of flow cytometry and quantitative analysis, no significant difference in cell apoptosis rates were observed between the BG- and Mo-extract groups when the dilution ratios were lower than 1/4 (Fig. 4d, e).

### Mo-extract inhibited the differentiation of osteoclast progenitors

**Fig. 5:**
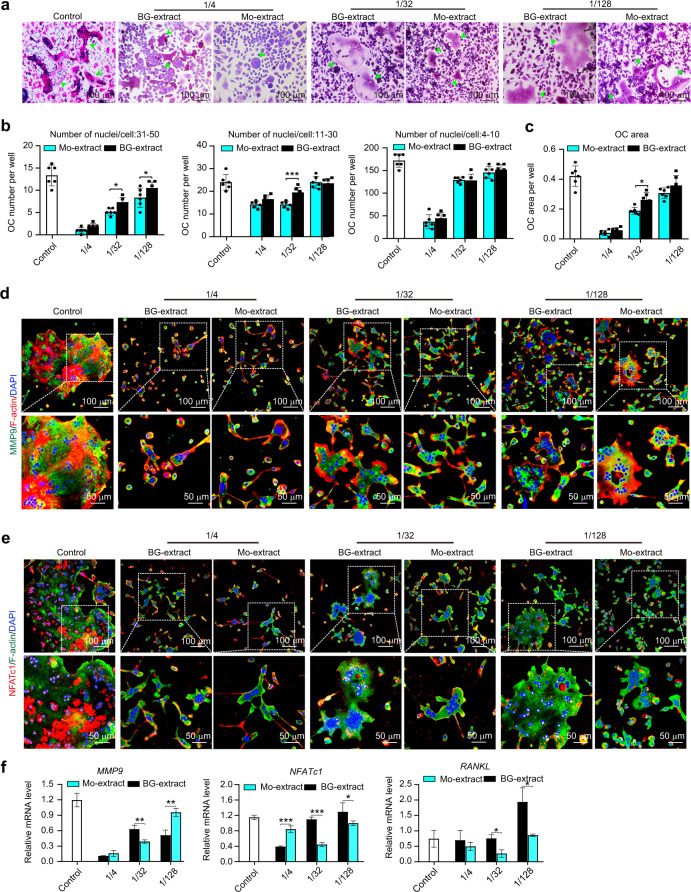
Effects of graded concentrations of diluted solutions of bioactive glass ceramic powder extracts with or without molybdenum (Mo-extracts or BG-extracts, respectively) on the differentiation of osteoclast progenitors; the concentrations of diluted BG- or Mo-extract solutions were set as 1/4, 1/32, or 1/128 (extract solution/total medium volume), and culture medium (control) was set as the negative control. **a** Osteoclastic enzymatic activity of osteoclast progenitors following incubation in graded concentrations of diluted BG- or Mo-extract solutions. Green arrows indicate TRAP-positive osteoclasts. Scale bar = 100 µm. **b** Quantitative analysis of osteoclasts containing 31–50, 11–40 or 4–10 nuclei based on optical images (*n* = 6). **c** Quantitative analysis of osteoclast area per well (*n* = 6). **d** Representative immunofluorescence images showing a specific marker (MMP9) of osteoclastic cells following incubation in graded concentrations of diluted BG- or Mo-extract solutions; MMP9 (a specific marker for osteoclasts) was labeled in green; F-actin rings were analyzed via phalloidin staining (red); nuclei were stained with DAPI (blue). Scale bar = 50 µm. **e** Representative immunofluorescence images showing a specific marker (NFATc1) for osteoclastic cells following incubation in graded concentrations of diluted BG- or Mo-extract solutions; NFATc1 (a specific marker for osteoclasts) was labeled in red; F-actin rings were analyzed via phalloidin staining (green); nuclei were stained with DAPI (blue). Scale bar = 50 µm. **f** Relative mRNA expression levels (normalized to *β-actin*) of osteoclastic differentiation-related genes (*MMP9*, *NFATc1* and *RANKL*) in cells following incubation in graded concentrations of diluted BG- or Mo-extract solutions (qRT–PCR assay, *n* = 3). The data are shown as the mean ± SD; *P < 0.05, **P < 0.01 and ***P < 0.001 indicate significant differences between the indicated columns

The effects of diluted solutions (dilution ratio: 1/4, 1/32, or 1/128) of BG- or Mo-extracts on differentiation of osteoclast progenitors were evaluated via TRAP staining (Fig. 5a). After 5 days of osteoclast induction, all osteoclast progenitors incubated in diluted solutions (dilution ratio: 1/4, 1/32, or 1/128) of either BG- or Mo-extract were found to form multinucleated and TRAP-positive osteoclasts, although incubation with both powder extracts appeared to decrease cellular osteoclastic differentiation compared to that observed after normal incubation (control). As the dilution ratios of BG- or Mo-extract decreased from 1/4 to 1/128, the number and size of multinucleated and TRAP-positive osteoclasts significantly increased; a 1/4 dilution of either BG- or Mo-extract led to notable inhibition of osteoclast formation (Fig. 5a). Quantitative analysis of TRAP-stained cells further demonstrated that the number of osteoclasts in diluted (dilution ratio: 1/4, 1/32, or 1/128) BG- or Mo-extract solutions decreased in a dose-dependent manner. When cells incubated with BG- or Mo-extract at the same dilution ratio were compared, Mo-extract at a 1/32 or 1/128 dilution ratio exerted a more significant inhibitory effect by decreasing the formation of mature osteoclasts containing 31–50 or 11–40 nuclei (Fig. 5b). Consistent with this finding, quantitative analysis of the area of osteoclasts indicated that increasing the dilution ratio of either BG- or Mo-extract decreased the area of osteoclasts, and a 1/32 dilution ratio of Mo-extract exhibited a much stronger inhibitory effect on the area of osteoclasts than the same dilution ratio of BG-extract (Fig. 5c). In parallel to TRAP staining, the formation of the F-actin ring, a specific actin structure important for bone resorption by active osteoclasts,^17^ and the expression of osteoclastogenesis-related proteins (MMP9 and NFATc1) and genes (*MMP9*, *NFATc1* and *RANKL*) were determined by immunofluorescence staining and qRT–PCR, respectively. Double immunofluorescence staining for MMP9 plus F-actin showed a higher MMP9 intensity in the plasma and a smaller actin ring in cells that underwent Mo-extract incubation compared to those incubated with BG-extract at the same dilution ratio, although both powder extracts at dilutions of 1/4, 1/32 or 1/128 exhibited inhibitory effects on the differentiation of osteoclast progenitors (Fig. 5d). Similarly, compared to 1/128 dilutions of BG- or Mo-extract, 1/4 and 1/32 dilutions of BG- or Mo-extract significantly reduced the expression levels of NFATc1 and the size of the actin ring, respectively; osteoclasts in culture medium showed the highest osteoclastogenesis-related protein expression (MMP and NFATc1) and the largest F-actin rings, and Mo-extract at all of the designated dilutions exerted stronger inhibitory effects than the BG-extract at the same dilutions (Fig. 5e). In line with the immunofluorescence staining results, qRT–PCR analysis indicated that diluted solutions (dilution ratio: 1/4, 1/32, or 1/128) of BG- or Mo-extract significantly decreased the expression levels of osteoclastogenesis-related genes (*MMP9*, *NFATc1* and *RANKL*) in a dose-dependent manner. Significantly decreased expression levels of all tested osteoclast-related genes were observed for 1/32 or 1/128 dilutions of Mo-extracts compared to solutions of BG-extracts at the same dilution ratios (Fig. 5f).

### Mo ions in the culture medium inhibited the differentiation of osteoclast progenitors

**Fig. 6:**
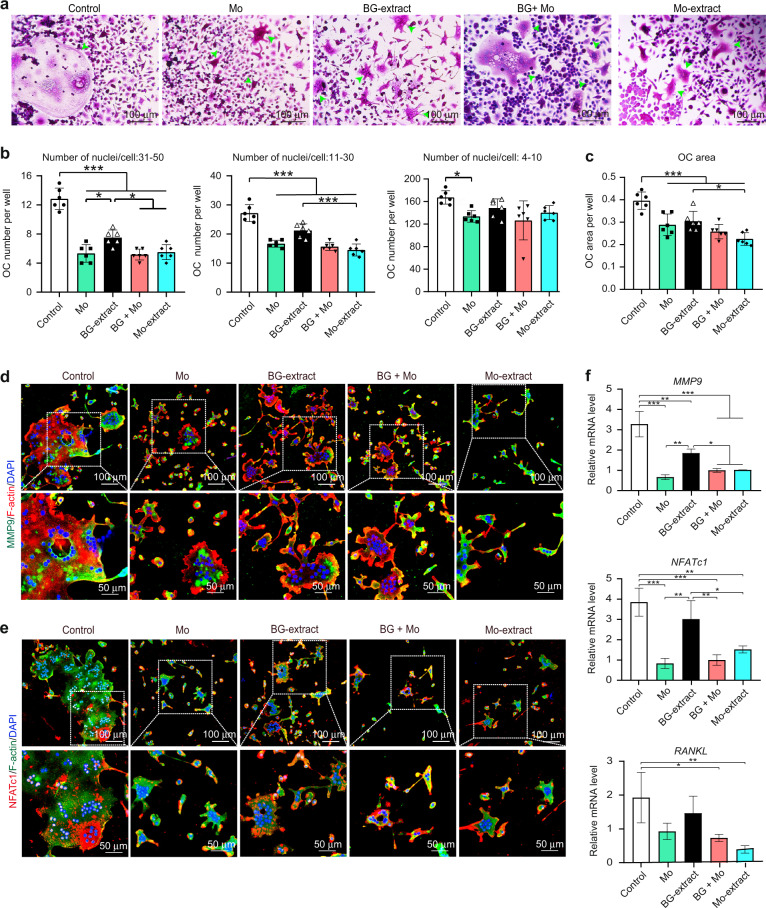
Effects of the presence of Mo ions in culture medium on the differentiation of osteoclast progenitors; culture medium containing Mo ions (Mo) and a diluted solution (dilution ratio: 1/32) of bioactive glass ceramic powder extract containing Mo ions (BG + Mo) were set as the experimental groups, while culture medium (Control), a diluted solution (dilution ratio: 1/32) of bioactive glass ceramic powder extract (BG-extract) and a diluted solution (dilution ratio: 1/32) of Mo-containing bioactive glass ceramic powder extract (Mo-extract) were set as the controls. **a** Osteoclastic enzymatic activity of osteoclast progenitors following incubation in different solutions. Green arrows indicate TRAP-positive osteoclasts. Scale bar = 100 µm. **b** Quantitative analysis of osteoclasts (indicated by green arrows) containing 31–50, 11–40 or 4–10 nuclei based on optical images (*n* = 6). **c** Quantitative analysis of osteoclast area per well (*n* = 6). **d** Representative immunofluorescence images showing a specific marker (MMP9) for osteoclasts following incubation in different solutions; MMP9 (a specific marker for osteoclasts) was labeled in green; F-actin rings were analyzed via phalloidin staining (red); nuclei were stained with DAPI (blue). Scale bar = 50 µm. **e** Representative immunofluorescence images showing a specific marker (NFATc1) for osteoclasts following incubation in different solutions; NFATc1 (a specific marker for osteoclasts) was labeled in red; F-actin rings were analyzed via phalloidin staining (green); nuclei were stained with DAPI (blue). Scale bar = 50 µm. **f** Relative mRNA expression levels (normalized to *β-actin*) of osteoclastic differentiation-related genes (*MMP9*, *NFATc1* and *RANKL*) in cells following incubation in different solutions (qRT–PCR assay, *n* = 3). The data are shown as the mean ± SD; *P < 0.05, **P < 0.01 and ***P < 0.001 indicate significant differences between the indicated columns

To further identify the role of Mo ions in the culture medium on the differentiation of osteoclast progenitors, we evaluated the cellular responses of osteoclast progenitors incubated in culture medium containing Mo ions or diluted (dilution ratio, 1/32) BG-extract solutions containing Mo ions. Compared to culture medium or the diluted solution of BG-extract, either culture medium containing Mo ions or the diluted solution of BG-extract containing Mo ions exerted more obvious inhibitory effects on the differentiation of osteoclast progenitors, indicated by the number and size of multinucleated and TRAP-positive osteoclasts observed by TRAP staining (Fig. 6a). Quantitative analysis demonstrated that the addition of Mo ions to either culture medium or a diluted solution of BG-extract significantly decreased the formation of mature osteoclasts containing 31–50 or 11–40 nuclei (Fig. 6b). Consistently, compared to culture medium alone, the presence of Mo ions in culture medium also significantly decreased the area of osteoclasts (Fig. 6c). In parallel to TRAP staining, double immunofluorescence staining for MMP9, NFATc1 and F-actin showed that the addition of Mo led to a lower MMP9 intensity in the plasma, a lower NFATc1 intensity in nuclei and a smaller actin ring size in the culture media (Fig. 6d, e). Again, the qRT–PCR analysis indicated that Mo ions in both culture media significantly decreased the expression of osteoclastogenesis-related genes (*MMP9*, *NFATc1* and *RANKL*). In all tests, culture medium or diluted solutions of BG-extract with Mo ions resulted in cellular responses similar to those observed with Mo-extracts (Fig. 6).

### Mo ions in the culture medium exerted inhibitory effects on ROS production and mitochondrial biogenesis in osteoclasts

**Fig. 7:**
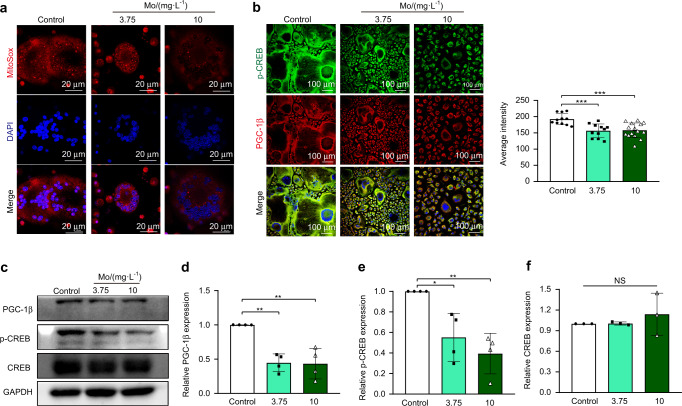
Effects of the presence of Mo ions in culture medium on mitochondrial biogenesis and ROS production in osteoclasts; culture medium containing 3.75 mg·mL^−1^ or 10 mg·mL^−1^ Mo ions (Mo) served as the experimental groups, while culture medium (Control) without Mo supplementation was set as the control group. **a** Representative immunofluorescence images showing ROS production in osteoclasts and quantitative analysis of the fluorescence intensity measured with ImageJ software (*n* = 11–15). Scale bar = 100 µm. **b** Representative immunofluorescence images showing PGC-1β and p-CREB expression in osteoclasts following incubation in different solutions; p-CREB (regulator of mitochondrial biogenesis) is labeled in green; PGC-1β (specific marker of mitochondrial biogenesis) is labeled in red; nuclei were stained with DAPI (blue). Scale bar = 100 µm. **c** Representative bands of mitochondrial biogenesis-related proteins (PGC-1β, p-CREB and CREB) in osteoclasts following incubation in different solutions (Western blot assays). **d**–**f** Quantitative analysis of PGC-1β (**d**) p-CREB (**e**) and CREB (**f**) expression in cells following incubation in different solutions (*n* = 3–4). The data are shown as the mean ± SD; *P < 0.05, **P < 0.01 and ***P < 0.001 indicate significant differences between the indicated columns

Following a 5-day osteoclastic induction, the effects of Mo ions on ROS production was analyzed using MitoSOX staining, while the expression of PGC-1β and p-CREB was analyzed via immunofluorescence staining and Western blot assays. The presence of Mo ions in culture medium at a concentration of either 3.75 mg·L^−1^ (3.75) or 10 mg·L^−1^ (10) significantly decreased mitochondrial ROS production in osteoclasts (Fig. 7a). Double-immunofluorescence staining showed that culture medium containing Mo ions decreased the PGC-1β and p-CREB staining intensity in osteoclasts in a concentration-dependent manner (Fig. 7b). Consistently, Western blot assays indicated that either 3.75 mg·L^−1^ or 10 mg·L^−1^ of Mo ions present in culture medium significantly decreased the expression levels of both mitochondrial biogenesis-related proteins (PGC-1β and p-CREB) in cells (Fig.7c–e), but the presence of Mo ions had no significant influences on the CREB expression in Western blot assays (Fig.7f).

### Mo-scaffold transplantation modulated in vivo osteogenesis/osteoclastogenesis to enhance alveolar bone regeneration

**Fig. 8:**
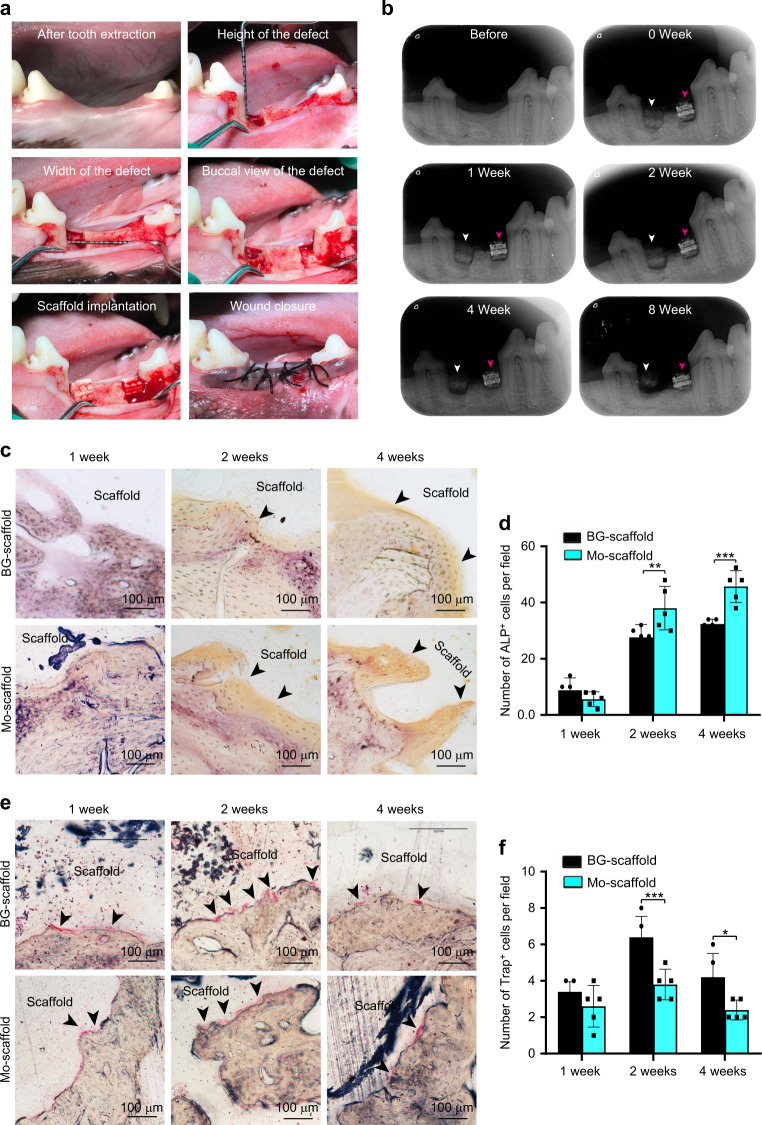
During the alveolar bone wound-healing cascade, implantation of bioactive glass ceramic scaffold with molybdenum (Mo-scaffold) led to increased osteogenesis and decreased osteoclastogenesis across alveolar bone defects in canines; implantation of bioactive glass ceramic scaffold without molybdenum (BG-scaffold) was used as the control. **a** Representative images showing the creation of critical-size alveolar bone defects at the tooth extraction site and the implantation of BG- or Mo-scaffold in canines. In both jaw quadrants, two one-wall intrabony defects with a size of 4 cm × 5 cm (width × height) were created at the distal site of the second mandibular premolar and at the mesial site of the fourth mandibular premolar, and then, the two defects were randomly subjected to transplantation of anatomically matched BG- and Mo-scaffolds. **b** Representative X-ray images showing bone defects subjected to transplantation of a BG-scaffold (white arrows) or Mo-scaffold (red arrows) at 0, 1, 2, 3, and 8 weeks post-surgery. **c** Representative immunohistochemical staining images showing the expression of the marker ALP by osteoblasts at the edge of the newly formed bone. **d** Semiquantitative analysis of ALP-positive cells per field based on immunohistochemical staining images (*n* = 5). **e** Representative immunohistochemical staining images showing the expression of the marker TRAP by osteoclasts at the edge of the newly formed bone. **f** Semiquantitative analysis of the TRAP-positive cells (black arrow) per field based on TRAP staining images (*n* = 5). The data are shown as the mean ± SD; *P < 0.05, **P < 0.01 and ***P < 0.001 indicate significant differences between the indicated columns

Throughout the animal experimental period, all canines tolerated the surgery quite well, and no discomfort in response to the transplants occurred; hence, no canines were excluded from the study. At 8 weeks after tooth extraction, the tooth sockets of the animals had all recovered and were well suited for alveolar bone defect creation. Following the placement of the scaffolds into the freshly created intrabony defects, both the BG- and Mo-scaffolds anatomically matched the defects well (Fig. 8a). When the gingival tissues covering the transplants were harvested for analysis at 2 weeks post-surgery,^27^ Mo-scaffolds were found to decrease the local mitochondrial ROS production (Fig. S2). During the alveolar bone wound-healing cascade, X-ray examination was used to track the remaining transplants and evaluate their stability within the defects. It was found that the Mo-scaffold transplants were tightly bonded with the defects at 1, 2, 4, and 8 weeks post-surgery, but in some cases, the BG-scaffold transplants had gradually separated from the bottom of defects (Fig. 8b).

When ALP and TRAP staining were used to analyze the effects of BG-/Mo-scaffold transplantation on regulation of the balance between osteogenesis and osteoclastogenesis in the alveolar bone defects at 1, 2 and 4 weeks post-surgery, ALP-positive cells were more frequently observed in bone defects receiving Mo-scaffold transplants than in bone defects receiving BG-scaffold transplants, although the number of ALP-stained cells at both material-tissue interfaces appeared to increase in a time-dependent manner (Fig. 8c). Further quantitative analysis demonstrated that the number of ALP-positive cells arising in response to Mo-scaffold transplantation was significantly greater than that arising in response to BG-scaffold transplantation at 2 and 4 weeks post-surgery (Fig. 8d). In parallel, after TRAP staining, a small number of TRAP-positive osteoclasts were observed at the material-tissue interfaces within defects receiving either BG- or Mo-scaffold transplants at 1 week post-surgery. Although the number of osteoclasts adjacent to all transplant surfaces appeared to increase from 1 to 2 weeks post-surgery and decrease from 2 to 4 weeks post-surgery, Mo-scaffold transplants resulted in a relatively lower rate of increase and a faster rate of decrease in the number of osteoclasts (Fig. 8e). Quantitative analysis of TRAP-positive osteoclasts indicated that at 2 or 4 weeks post-surgery, the number of osteoclasts at the edge of the material-tissue interfaces of Mo-scaffold transplants was much smaller than that at the edge of the material-tissue interfaces of BG-scaffold transplants (Fig. 8f).

**Fig. 9:**
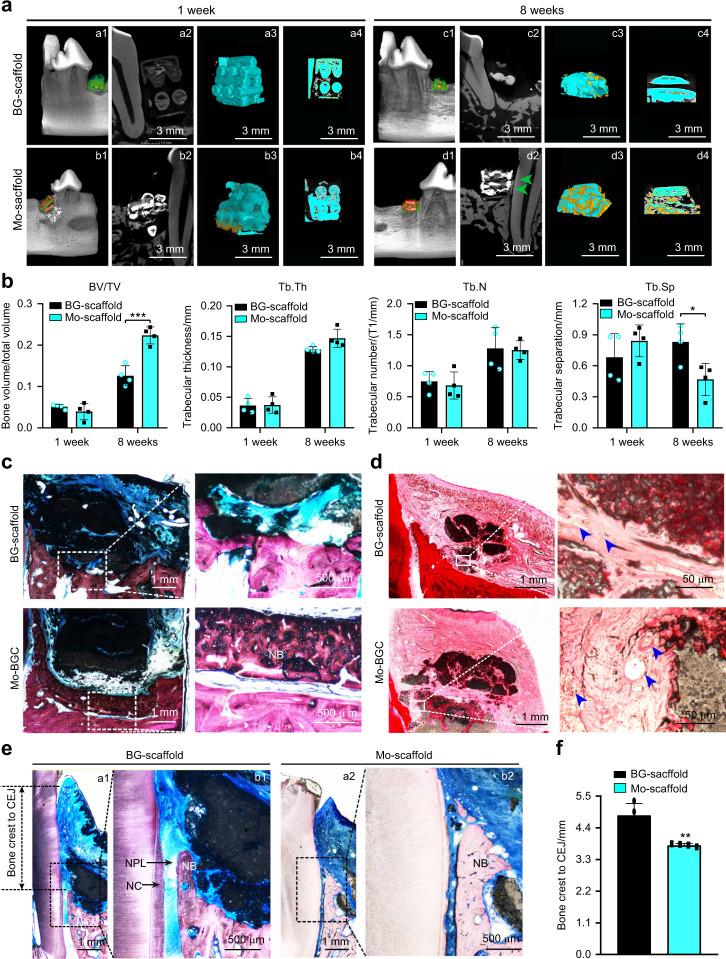
At 8 weeks post-surgery, implantation of a molybdenum-containing bioactive glass ceramic scaffold (Mo-scaffold) resulted in increased alveolar bone regeneration across alveolar bone defects in canines; implantation of a bioactive glass ceramic scaffold without molybdenum (BG-scaffold) was used as the control. **a** Representative micro-CT images showing the remaining implants and the newly formed bone within the bone defect region at 1 and 8 weeks post-surgery. (a1, b1, c1 and d1) Representative 3D reconstruction images showing the transplants within the alveolar bone defects at 1 and 8 weeks post-surgery. (a2, b2, c2 and d2) Representative cross-section view images showing the newly formed bone (green arrows) alongside the root and the Mo-scaffold transplants at 8 weeks post-surgery; (a3,b3, c3 and d3) Representative 3D reconstruction images showing the newly formed bone within the transplants (cyan for transplants and yellow for newly formed bone); (a4, b4, c4 and d4) Representative cross-section view images showing the newly formed bone growing into the transplants at 8 weeks post-surgery (cyan for transplants and yellow for newly formed bone). Scale bar = 3 mm. **b** Quantitative analysis of bone volume/total volume (BV/TV), trabecular thickness (Tb.Th), trabecular number (Tb.N) and trabecular separation (Tb.Sp) based on micro-CT scanning at 1 and 8 weeks post-surgery. **c** Representative methylene blue-acid fuchsin staining images showing the newly formed bone surrounding transplants within the alveolar bone defect region at 8 weeks post-surgery. Left panel: overview of the alveolar bone defect region, scale bar = 1 mm; right panel: magnified view showing the newly formed bone on the material-tissue interfaces (marked area from the left panels), scale bar = 500 µm. **d** Representative H&E staining images showing the newly formed vessels surrounding transplants within the alveolar bone defect region at 8 weeks post-surgery. Left panel: overview of the bone defect region, scale bar = 1 mm; right panel: magnified view showing the newly formed vessels on the material-tissue interfaces (marked area from the left panels), scale bar = 50 µm. **e** Representative methylene blue-acid fuchsin staining images showing the newly formed hybrid periodontal tissues in the defects, including new bone (NB), new periodontal ligament (NPL) and new cementum (NC), in canines at 8 weeks post-surgery. (a1-a2) Representative images of methylene blue-acid fuchsin staining showing the newly formed bone-PDL-cementum complex in created defects, scale bar = 1 mm; (b1-b2) magnified view showing the NPL (marked area from the left panels), scale bar = 500 µm. **f** Histometric analysis of the height from the bone crest to the cemento-enamel junction (CEJ) site. Landmarks and parameters used for histometric analysis are shown in (**e**), and the data are shown as the mean ± SD (*n* = 4); *P < 0.05, *P < 0.01 and ***P < 0.001 indicate significant differences between the indicated columns

The bone regeneration outcomes of canine alveolar bone defects receiving Mo- or BG-scaffold transplants were evaluated by micro-CT, methylene blue-acid fuchsin staining and hematoxylin-eosin (H&E) staining. At 1 week post-surgery, micro-CT scanning showed that both BG- and Mo-scaffold transplants anatomically matched the alveolar bone defects well, and at this stage, little regenerated bone (yellow) within the scaffolds (cyan) could be observed. At 8 weeks post-surgery, larger amounts of newly formed bone (labeled with green arrows) alongside the tooth root and the transplants (cyan) could be observed in defects receiving Mo-scaffold transplants, with obvious newly formed bone (yellow) growing into the inner compartments of the scaffolds (Fig. 9a). Quantitative micro-CT analysis showed no significant difference in BV/TV, Tb.Th, Tb.N or Tb.Sp between the Mo- and BG-scaffold groups at 1 week post-surgery. At 8 weeks post-surgery, BV/TV was significantly increased and the Tb.Sp was decreased in alveolar bone defects receiving Mo-scaffold transplantation compared to those receiving BG-scaffold transplantation (Fig. 9b). When the hard tissue slices across the bone defect were analyzed by methylene blue-acid fuchsin staining at 8 weeks post-surgery, large amounts of newly formed bone tissue at the material-tissue interface could be observed in defects receiving the Mo-scaffold transplants (Fig. 9c). As revealed by H&E staining, more new vessels were formed at the material-tissue interfaces of Mo-scaffold transplants than those of BG-scaffold transplants (Fig. 9d). Although newly formed hybrid periodontal tissues, including bone, PDL and cementum, were observed in defects receiving either BG- or Mo-scaffold transplants, it appeared that much more bone formed alongside the tooth root in defects receiving Mo-scaffold transplants, and substantial new PDL tissue was found anchored into the neo-cementum and bone (Fig. 9e). Histometric analysis further indicated that the height from the bone crest to the cemento-enamel junction (CEJ) site in the Mo-scaffold group was significantly smaller than that in the BG-scaffold group, suggesting more alveolar bone regeneration in defects receiving Mo-scaffold transplants (Fig. 9f).

## Discussion

Periodontitis is one of the most common chronic inflammatory diseases in humans and affects over 45%–50% of the global adult population to various extents.^32,33^ Alveolar bone loss is a common consequence of periodontitis and a major cause of periodontitis-induced tooth loss.^34,35^ Although some progress has been made recently, the regrowth of damaged alveolar bone around diseased teeth is still a substantial challenge in the field of regenerative dentistry.^12,36^ In periodontitis, the local inflammatory response significantly inhibits osteoblastic bone production while promoting osteoclastic bone resorption, which ultimately leads to irreversible bone loss.^7,8^ In this context, the positive regulation of osteogenesis plus the negative feedback regulation of osteoclastogenesis may be a reliable way to achieve alveolar bone regeneration. However, typically, comodulating osteogenesis and osteoclastogenesis is difficult due to the distinct activities of these two coupled events.^37^ For example, the addition of growth factors and osteogenic inducers to bone repair materials can promote osteogenesis, but such a design exerts little effect on osteoclastogenesis.^38^ Bisphosphonates have been used clinically to prevent bone resorption, but they have been found to simultaneously impair osteoblast activity.^39^ Bioactive factors and therapeutic agents have also been demonstrated to promote alveolar bone regeneration in vivo, but their therapeutic use in clinics has been limited by their short half-life, suboptimal biodistribution, and off-target side effects, such as tumorigenesis, diarrhea, and constipation, following long-term use.^17,40,41^ Advanced release technologies have already been incorporated into cell-instructive scaffolds that can deliver bioactive factors or drugs to target sites according to the desired dose, timing, and sequence, but for clinical use, biomaterials must be not only efficacious for therapeutic use but also cost-effective for production.^41–46^ Hence, there is an urgent need to develop simple, safe and practical platforms that are able to orchestrate the balance of osteogenesis and osteoclastogenesis in bone tissue engineering and regeneration strategies.^47,48^ Although using biomaterials to improve osteogenesis has been successful in many published studies, the negative feedback regulation of osteoclastic activity among the distinct and coupled activities of osteoblasts/osteoclasts remain largely unexplored.^12,13^

Given the multifunctional effects of bioactive ions reported to comodulate the pro-osteogenic and anti-osteoclastogenic healing processes^18,20,49–51^ and the previously reported role of Mo in promoting periodontal regeneration,^29^ in this study, we further investigated the therapeutic effect of Mo ions from the standpoint of orchestrating the cellular responses of osteoblasts and osteoclasts using in vitro and in vivo models. Previously, we found that a strut-packed Mo-containing bioactive glass ceramic scaffold with a hollow-pipe structure can promote periodontal wound healing;^29^ however, the hollow-pipe structure can cause unnecessary infection due to the poor filling of newly formed bone.^52^ To overcome this drawback, a similar Mo-containing bioactive glass ceramic scaffold with a solid-pipe structure was successfully printed in the present study. Bioactive glass ceramic was used as a substrate in our series of experiments due to its osteoconductive, osteoproductive and osteoinductive characteristics.^53,54^ Based on a sol-gel method and 3D-printing technology, Mo was successfully incorporated into the bioactive glass ceramic scaffold. Compared to the scaffold without Mo incorporation, Mo incorporation was found to have no influence on the appearance and overall topographic characteristics of the scaffold (Fig. 1a and b), although there was slight difference in the surface microstructure between the BG- and Mo-scaffolds (Fig. 1b). However, Mo incorporation significantly changed the degradation behavior and ion release profiles of the scaffolds (Fig. S1); when Mo was introduced into the bioactive glass ceramic scaffold, it reacted with O and Ca to form the CaMoO_4_ phase, which can melt and form connections to other phases, such as SiO_2_, CaSiO_3_ and Ca_3_(PO_4_)^2^, due to its low melting temperature point (965 °C), making the scaffold more compact.^28^ The increased intensities and decreased micropores reduced the contact area between the scaffold and solution, thus significantly inhibiting degradation and ion release.^28,29^

The bioactivity to bioceramic scaffolds is largely determined by their incorporated ions. To interrogate cellular response to ions released from the synthesized scaffolds, we prepared extracts derived from Mo- and BG-scaffold powders (Mo- and BG-extracts) and diluted them in culture medium for in vitro cell incubation. We investigated the powder extracts instead of the scaffold extracts because a fast release of ions can be achieved from powders compared with that from the synthesized scaffolds.^28,29,55,56^ One important result of the present study is that the prepared powder extracts exerted positive effects on the proliferation and osteogenic differentiation of BMMSCs. CCK-8 assays indicated that both BG-extracts and Mo-extracts promoted the proliferation of BMMSCs. However, the proliferative capacity of BMMSCs did not increase in a dose-dependent manner following incubation in graded dilute solutions of powder extracts. This finding was consistent with previously published studies showing that 0.1 mmol·L^−1^ Si (2.8 mg·L^−1^) could exert more powerful effects on proliferation of C2C12 myoblast cells than 0.5 mmol·L^−1^ (14.0 mg·L^−1^) or 1.0 mmol·L^−1^ (28.0 mg·L^−1^) Si ions.^57^

When the effects of the BG- and Mo-extracts on cell osteogenic differentiation were explored, Alizarin Red S staining showed that 1/32 BG- and Mo-extracts enhanced the osteogenic differentiation (Fig. 2a). The bioactive effects of BG-extracts were mainly due to the release of Si ions, whose pro-osteogenic effects are closely associated with their concentrations and the stem cell type. For example, previous studies have indicated that 1/4 akermanite extracts provide greater enhancement of the osteogenic potential of human adipose-derived stem cells than 1/2 akermanite extract;^58^ 1/8 bioactive glass ceramic extract showed stronger promotion of the osteogenic potential of human bone marrow mesenchymal stem cells than 1/2 bioactive glass ceramic extract.^28^ In the present study, Alizarin Red S staining and quantitative analysis showed that BG-extracts enhanced osteogenesis in mouse BMMSCs compared with the control group (Fig. 4a). However, cells exposed to 1/4 BG-extracts did not show the strongest osteogenic capacities, perhaps due to the concentration of bioactive ions and the cell type. Furthermore, ALP staining and Alizarin Red S staining showed that Mo-extracts significantly increased the osteogenic differentiation of BMMSCs (Fig. 2b–e). These findings were consistent with our previous finding that the ions released from Mo-scaffolds could meet the long-term ion demands of stem cell differentiation throughout the period of cell incubation.^29^ However, due to the complex ionic composition of Mo-extracts and BG-extracts, such as the different Si and Mo ion concentrations, and because Si can also exert positive effects on the osteogenic differentiation of stem cells,^20,21,49,59^ determination of the roles of Mo ions in the positive effects of Mo-extracts still requires further research. After supplementing culture medium and BG-extracts with Mo ions (the same concentration as that in the Mo-extract cultures), we found that Mo ions alone can promote osteogenesis. Consistently, addition of Mo ions to BG-extracts significantly enhanced the pro-osteogenic effects of BG-extracts and exerted effects similar to those of Mo-extracts (Fig. 3). Taken together, these data indicate that Mo ions can significantly enhance the osteogenic potential of stem cells and that the Mo-extract-enhanced osteogenesis in vitro was mainly due to the Mo ions present in the powder extract. Additionally, the results did not indicate that Mo could enhance osteogenesis in a dose-dependent manner. Actually, most bioactive ions have suitable concentrations, and high concentrations may damage cell functions. Our previously published study indicated that 2–6 mg·L^−1^ of Mo ions showed the strongest promotion of osteogenesis,^28^ which can explain why 1/32 Mo-extracts (the concentration of Mo is ((3.75 ± 0.33) mg·L^−1^) showed more powerful pro-osteogenic effects than 1/4 Mo-extracts (the concentration of Mo is ((31.65 ± 0.57) mg·L^−1^).

In addition to positive regulation of bone-forming osteoblast activity, negative feedback regulation of osteoclastic activity is equally important for modulating the bone healing cascade, including but not limited to reducing bone resorption, which results in successful bone regeneration.^5,45^ Although many ions have been demonstrated to endow bone regenerative biomaterials with more powerful osteogenic effects and some of them have been found to modulate osteoclastogenesis via their osteoimmunomodulatory activity,^60,61^ little is known about their direct influence on osteoclastogenesis. To answer this question, the effects of Mo-extracts on the differentiation of osteoclast progenitors were further explored. Cell seeding experiments showed that Mo-scaffolds supported the attachment and growth of osteoclast progenitors, as demonstrated by SEM and live/dead staining (Fig. 4a–c). We also found that Mo-extracts at a dilution ratio less than 1/4 exerted no effects on cell apoptosis (flow cytometry assay; Fig. 4d, e). Consistent with our previous findings,^28,29^ these data demonstrate the good cytocompatibility of Mo-scaffolds with osteoclast progenitors. To investigate the effects of Mo-extracts on the differentiation of osteoclast progenitors, TRAP staining, immunofluorescence staining and qRT–PCR analysis indicated that both BG- and Mo-extracts inhibited osteoclastic differentiation in a dose-dependent manner. Compared to BG-extracts, Mo-extracts exhibited more robust inhibitory effects (Fig. 5a–f). Here, the inhibitory effects of BG-extracts on osteoclastic differentiation may be due to the released Si ions because their anti-osteoclastic capacity has been demonstrated previously.^62^ Considering that the concentration of Si ions in Mo-extracts was much lower than that in BG-extracts, the enhanced anti-osteoclastic effects of Mo-extracts must arise from other ions in the extracts, such as Mo. The use of Mo ions in cell medium or BG-extracts may answer this question. As expected, our data indicated that Mo ions in medium at a concentration equivalent to that in Mo-extracts exerted inhibitory effects similar to those of Mo-extracts on the differentiation of osteoclast progenitors, and Mo addition significantly strengthened the anti-osteoclastic properties of BG-extracts (Fig. 6a–f). Therefore, these data verified that Mo ions can directly inhibit the differentiation of osteoclast progenitors and that the anti-osteoclastic effects of Mo-extracts in vitro mainly arise from the Mo ions contained in the extracts.

Osteoclast differentiation is a high energy-demand process involving facile migration and rapid secretion of protons or protein-degrading enzymes.^30,63^ Indeed, osteoclasts are widely known as mitochondria-rich cells, and the number and size of mitochondria increase robustly during the phenotypic changes from mononuclear precursor cells to multinuclear osteoclasts.^64,65^ Mitochondrial biogenesis requires the orchestrated synthesis of nuclear and mitochondria-encoded DNA, proteins, and mitochondrial membranes, and biogenesis of these organelle networks is vital for their inherent functions.^63,64^ As the master regulator of mitochondrial biogenesis, PGC-1β has been reported to play a fundamental role in osteoclastic differentiation and function.^30,63^ For example, RANKL can significantly upregulate PGC-1β expression via CREB through ROS production,^30^ and knockout of PGC-1β in mice can increase bone mass by inhibiting osteoclast function.^30,66^ Previously, we found that Mo ions can accumulate in mitochondria to influence mitochondrial function in macrophages.^29^ Given that osteoclasts are derived from monocyte-/macrophage-lineage cells, we hypothesized that the inhibitory effects of Mo ions on osteoclastic differentiation are due to their effects on mitochondrial biogenesis. The addition of Mo ions in culture medium was found to significantly decrease mitochondrial ROS production in osteoclasts (Fig. 7a). Meanwhile, Mo ions also significantly decreased the p-CREB and PGC-1β expression levels, demonstrated by immunofluorescence staining and Western blotting results (Fig. 7b–f). In vivo MitoSOX staining also indicated that Mo-scaffolds decreased mitochondrial ROS production. These findings strongly indicated that Mo ions can inhibit mitochondrial biogenesis in osteoclasts and that the anti-osteoclastic effects of Mo ions may be due to their ability to inhibit mitochondrial biogenesis and ROS production.

The other interesting result was that the 3D-printed Mo-scaffolds can orchestrate the action of osteoblasts and osteoclasts simultaneously to benefit bone regeneration in vivo. For verification of the pro-osteogenic and anti-osteoclastic effects of Mo-scaffolds in vivo, both the BG- and Mo-scaffolds were transplanted into a clinically relevant alveolar bone defect, similar to the models we used previously^29^ (Fig. 8a), and a slow degradation rate of the Mo-scaffold was observed in vivo (Fig. 8b). For both BG- and Mo-scaffolds, the activity of osteoclasts (labeled by TRAP) increased rapidly and peaked at 2 weeks post-surgery, while the activity of osteoblasts (with labeled ALP) increased gradually within 4 weeks following scaffold transplantation (Fig. 8c–f), suggesting that early activation of osteoclasts occurs during periodontal healing.^67^ TRAP and ALP staining observations indicated that Mo-scaffold transplantation resulted in a significantly decreased number of osteoclasts and an increased number of osteoblasts compared to BG-scaffold transplantation (Fig. 8c–f). These results indicate that the Mo-scaffold can rebuild a more bioactive microenvironment than the BG-scaffold to orchestrate osteogenesis and osteoclastogenesis in vivo. Although this study is not the first to use bioactive ions to promote osteogenesis while suppressing osteoclastogenesis,^9,20–22^ we are the first to demonstrate a synergistic effect of Mo ions on the regulation of osteogenesis and osteoclastogenesis. Considering that Mo ions can finely tune macrophages towards the M2 phenotype and that M2 macrophages can promote osteogenesis while inhibiting osteoclastogenesis by secreting IL-10,^68^ we believe that Mo can induce pro-osteogenic and anti-osteoclastogenic cellular responses in collaboration with macrophages.

The periodontium is a highly hierarchical organ where intercalated periodontal ligament fibers insert into the hard cementum and alveolar bone. The complex architecture of the periodontium makes it one of the most difficult tissues to repair.^69^ In the past, various types of scaffolds have been developed to reconstruct periodontal defects, such as biphasic, triphasic, and multiphasic scaffolds. These scaffolds succeeded in mimicking the architectural and compositional features of the periodontium but could not reproduce the natural structure and properties of hybrid periodontal tissues.^45,46,69^ Moreover, most of these recent developments are still in their infancy, and widespread clinical translation is impeded by the complexity of scaffold design.^13,70,71^ In the present study, critical-size alveolar bone defects in canines, which can provide adequate space and a regular configuration to secure scaffold transplantation, were used to evaluate the therapeutic effects of Mo-scaffolds due to their resemblance to alveolar bone defects in humans.^71^ However, the surgically created defects differ from periodontitis-induced defects in terms of pathogenesis, morphology and tissue histology. Both micro-CT scanning and histological staining indicated that the Mo-scaffold, as a monophasic biomaterial, succeeded in promoting the regeneration of the periodontal bone-ligament-cementum complex in canines (Fig. 9). Considering that a strut-packed Mo-containing bioactive glass ceramic scaffold with either a hollow-pipe structure (tested previously, see ref. ^29^) or solid-pipe structure (tested in the present study) exhibited the capacity to promote periodontal healing, we can conclude that the Mo-containing bioactive glass ceramic scaffolds play a crucial role in cellular modulation and hence contribute to the final regenerative outcome.

**Fig. 10:**
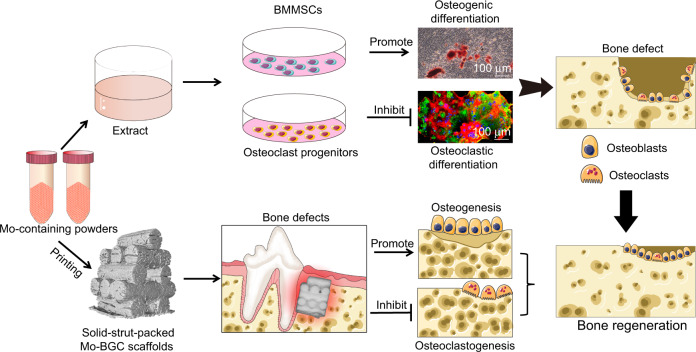
Schematic illustration of the study design and main findings of the current study. For cellular investigations, diluted solutions of extracts derived from the molybdenum (Mo)-containing scaffold-printing powders significantly promoted osteogenic differentiation of BMMSCs while inhibiting the differentiation of osteoclast progenitors. In a clinically relevant critical-size alveolar bone defect model, 3D-printed Mo-containing bioactive glass ceramic scaffold with solid-strut-packed structures (Mo-scaffold) exerted stronger function to enhance osteogenesis while inhibit osteoclastogenesis throughout the bone healing process, leading to enhanced alveolar bone regeneration

In addition to the previously reported immunomodulatory activity of Mo-containing bioactive glass ceramic scaffolds,^29^ the present study identified a new role of Mo-scaffolds in enhanced alveolar bone regeneration via orchestration of osteogenesis and osteoclastogenesis. In this context, the Mo-extracts were found to promote the osteogenic differentiation of BMMSCs while inhibiting the differentiation of osteoclast progenitors by scavenging ROS and inhibiting mitochondrial biogenesis in osteoclasts. Further study indicated that the addition of Mo ions to either cell medium or BG-extracts at the same levels presented in Mo-extract cultures exerted similar dual cellular functions. Consistent with the in vitro results, implantation of Mo-scaffolds led to an enhanced regenerative outcome in alveolar bone defects through positive regulation of the activity of bone-forming osteoblasts in combination with negative feedback regulation of osteoclastic activity (Fig. 10). In addition to participation in material-mediated immunomodulation, our study reveals for the first time the contribution of Mo ions to anti-osteoclastogenic cellular responses and, particularly, to the orchestration of the balance between osteoblasts and osteoclasts across the bone healing cascade and provides additional evidence supporting the use of Mo ions as multifunctional cellular modulators in future biomaterial designs.

## Materials and methods

### Synthesis of Mo-containing bioactive glass ceramic powder (Mo-powder)

The Mo-powder was synthesized using a sol-gel method as reported previously.^28,29^ Briefly, 157 mL of TEOS (tetraethoxysilane) and 25 mL of HNO_3_ (2 mol·L^−1^) were added into 200 mL of deionized water and stirred for 30 min. Then, 41.30 g of Ca(NO_3_)_2_⋅4H_2_O, 13.24 g of (NH_4_)_6_Mo_7_O_24_·4H_2_O and 8.53 mL of triethyl phosphate (TEP) were added at molar ratios of 70: 17.5: 5: 7.5 (Si: Ca: P: Mo), yielding Mo-powder. To prepared bioactive glass ceramic powder (BG-powder) without Mo incorporation, 59 g of Ca(NO_3_)_2_⋅4H_2_O and 8.53 mL of TEP were added at molar ratios of 70: 25: 5: 0 (Si: Ca: P: Mo). After stirring for 5 h, the precursor solution was transferred to an air oven at 60 °C for 24 h and 120 °C for 48 h. The dried bioactive glass gel was ball-milled for 5 h at 500 r·min^−1^ and then calcined at 800 °C for 3 h. The obtained Mo- and BG-powers were then sifted with a 300 mesh sifter.

### Preparation and characterization of Mo-scaffolds

To generate Mo- and BG-scaffolds, 20 g of Mo- or BG-powders with 30 g of photosensitive resin (Wanhao Co. Ltd., Ningbo, Zhejiang, China) were mixed to obtain a photosensitive slurry, and a digital light processing (DLP)-based 3D printer (AUTOCERA-M, Beijing Ten Dimensions Technology Co. Ltd., Beijing, China) was applied for scaffold printing. After being rinsed in alcohol and dried at room temperature overnight, the printed Mo- and BG-scaffolds were sintered at 1 300 °C with a heating rate of 2 °C/min for 3 h prior to use. Similar to methods we reported recently,^28,29^ the overall shape of the 3D-printed BG- and Mo-scaffolds was observed via optical microscopy (S6D, Leica Microsystems, Heidelberg, Germany), while the surface microstructure of both scaffolds was characterized by scanning electron microscopy (SEM; Hitachi S-4800; EIKO Engineering, Tokyo, Japan). The element mapping of the printed scaffolds was visualized via energy dispersive X-ray spectrometry (EDS; SU8220,Hitachi,Tokyo, Japan). The degradation and ion-releasing behaviors of BG- and Mo-scaffolds were analyzed based on previously published studies.^28,29^ Briefly, the testing scaffolds were immersed into tris-HCl solution (pH = 7.4) at a ratio of 200 mL·g^−1^ (tris-HCl solution volume/scaffold mass) (*n* = 4 for each group), and these specimens were put into an incubator shaker for 3, 7, 10, 14, 21, and 28 days at 37 °C. At each time point, the scaffolds were harvested and dried in an air oven at 60 °C for 24 h. The final weight of each scaffold was measured. In addition, the tris-HCl solutions were collected at each time point and the concentrations of Mo, Ca, Si, P ions were measured via inductively coupled plasma–mass spectrometry (ICP-MS; NexION™, PE, USA).

### Graded concentration of Mo-scaffold powder extracts

To investigate the biological effects of the Mo-scaffold on the osteogenic and osteoclastogenic differentiation of cells, we indirectly observed in vitro cellular responses to bioactive ions released from the printed scaffold observed using a dilute solution of Mo-extract as the cell culture medium; a dilute solution of BG-extract was set as the control. To prepare Mo-extract and BG-extract, 2.0 g of bioactive glass ceramic powder or Mo-containing bioactive glass ceramic powder was fully immersed in 10 mL of α-minimum essential medium containing L-glutamine, ribonucleic acid and deoxyribonucleic acid (complete medium; Invitrogen, Carlsbad, CA, USA) and shaken at 37 °C at a speed of 120 r·min^−1^. According to our previously published studies,^28,29^ the solution was centrifuged at 8000 r/min for 5 min after 24 h of shaking, and then, the supernatants were collected and filtered through 0.22-µm pore filters (Millipore, Billerica, MA, USA) to ensure sterility. The concentrations of Mo, Si, Ca and P in the BG-/Mo-extracts were analyzed via ICP–MS (NexIONTM 168; PerkinElmer, MA, USA) to ensure the sufficient release of ions form powders. For cell incubation, the filtered solution was diluted with culture medium at a ratio of 1/4, 1/32 or 1/128 (extract solution/total medium volume), resulting in 3 diluted solutions of BG- and Mo-extracts with graded concentrations representing a high medium and low concentration of ions, respectively.

### Effects of graded concentration of Mo-extracts on the proliferation and osteogenic differentiation of mouse primary BMMSCs

Mouse BMMSCs were obtained according to previously reported methods.^10^ The proliferation capacity of BMMSCs incubated in diluted solutions of BG-/Mo-extracts was analyzed using a Cell Counting Kit-8 (CCK-8) assay kit (Seven Sea Biotechnology, Shanghai, China). According to the manufacturer’s instructions, 1 × 10^3^ cells per well were incubated in a 96-well plate (Costar, Cambridge, MA, USA) supplemented with 200 µL of diluted solution (dilution ratios: 1/4, 1/32 and 1/128). Cells incubated in culture medium were used as the control. At a fixed timepoint during the 7-day test period, 20 µL of CCK-8 solution was added to each testing well, followed by a 3.5-h incubation at 37 °C. The supernatant was then transferred to another 96-well plate, and the optical density (OD) at 450 nm was immediately determined using a microplate reader (ELx800, BioTek Instruments, Highland Park, USA).To assess the effects of the diluted BG-/Mo-extract solutions on the osteogenic potential of BMMSCs, cells were seeded in 12-well culture plates at a density of 2 × 10^5^ cells per well. Considering that the supplemented osteogenic induction components can influence the concentration of ions by changing the compositions of the medium, the graded concentration solutions of Mo-scaffold powder extracts were first prepared, followed by supplementation with 50 μg·mL^−1^ vitamin C, 10 nmol·L^−1^ dexamethasone and 10 mmol·L^−1^ β-glycerophosphate (all purchased from Sigma–Aldrich, St. Louis, MO, USA). When the cells reached 80% confluence, the complete medium was changed to osteoinductive medium; all the media were refreshed every 3 days. Following a 7-day induction, the osteogenic differentiation of BMMSCs was analyzed according to the formation of calcified nodules (Alizarin Red S staining) and ALP activity following previously reported methods.^10^

### Effects of Mo ions in culture medium on the osteogenic differentiation of BMMSCs

To assess the effects of Mo ions on the osteogenic differentiation of BMMSCs, culture medium containing Mo ions was prepared as follows: 18.39 mg of (NH_4_)_6_Mo_7_O_24_·4H_2_O was dissolved in 10 mL of cell culture medium to obtain a 1 000 mg·L^−1^ Mo ion solution, and then, 1 000 mg·mL^−1^ Mo ion solution was used to prepare culture medium containing Mo ions. Because the concentration of Mo ions in the diluted solution of Mo-extract (dilution ratio: 1/32) was 3.75 mg·L^−1^ (see Supporting information), culture medium containing 3.75 mg·mL^−1^ of Mo ions (Mo) or a diluted solution of BG-extract (dilution ratio: 1/32) containing 3.75 mg·L^−1^ of Mo (BG + Mo) was used in the present study with the aim of assessing the influence of the presence of Mo ions in culture medium on osteogenic differentiation of cells. Culture medium (Control) and diluted solutions of BG-extract (dilution ratio: 1/32) were designed as the negative controls, and diluted solutions of Mo-extract (dilution ratio: 1/32) were used as the positive control. Similarly, the osteogenic differentiation of BMMSCs following a 7-day induction was analyzed in terms of Alizarin Red S staining and ALP activity. In parallel, the expression levels of osteogenesis-related proteins (Runx2 and BSP-1) and genes (*Runx2*, *ALP* and *SP7*) in BMMSCs were evaluated by Western blot^72^ and quantitative real-time polymerase chain reaction (qRT–PCR) analyses, respectively. The primary antibodies used in the Western blot assay in the present study were as follows: antibodies targeting BSP-II (1:1 000; A16220, ABclonal, Wuhan, Hubei, China), Runx2 (1:1 000; #12556, Cell Signaling Technology, Danvers, MA, USA) and β-actin (1:5 000; P20536-1-AP, Proteintech, Rosemont, IL, USA).Protein quantification was conducted using ImageJ Plus software (National Institute of Health, Bethesda, MD, USA), and the gray value of each target protein was normalized to that of β-actin before comparison.

### Ex vitro biocompatibility of Mo-scaffolds cultured with osteoclast progenitors

In addition to those of BMMSCs, the cellular responses of osteoclast progenitors to biomaterials also play crucial roles in the alveolar bone regeneration process. Therefore, we further investigated how the presence of Mo ions in the scaffold or culture medium affected the growth and differentiation of osteoclast progenitors. Mouse osteoclast progenitors were harvested and cultured according to our previously reported methods^11,73^ and used to assess the ex vivo biocompatibility of BG-/Mo-scaffolds with osteoclast progenitors. Cell adhesion, morphology, and viability were observed by seeding osteoclast progenitors onto BG-/Mo-scaffolds, and cell apoptosis was analyzed by incubating osteoclast progenitors in diluted solutions of BG-/Mo-extracts.

#### Cell adhesion, morphology and viability assays

Osteoclast progenitors (1 × 10^6^) were seeded onto the 3D-printed BG-/Mo-scaffolds and incubated in culture medium for 24 h. For cell adhesion and morphology, each sample was fixed in 2.5% glutaraldehyde (Sigma–Aldrich) overnight at 4 °C and then gradually dehydrated in ethanol solutions with gradient concentrations (10%, 20%, 40%, 60%, 80%, 90%, and 100%). Subsequently, the samples were critical-point dried using hexamethyldisilane (Sigma–Aldrich) at room temperature before sputter-coating with Pb/Au. Cell adhesion and morphology on both scaffolds were finally observed and compared using field-emission SEM (Hitachi S-4800).To assess cell viability, a Calcein-AM/PI Double-Stain Kit (Yeasen Biotechnology, Shanghai, China) was used according to the manufacturer’s instructions. Each sample was stained with 5 mL of PBS containing 15 μL of 200 calcein-AM and 5 μL of PI for 15 min in the dark. Then, each sample was rinsed and observed with a confocal laser microscope (A1 PLUS, Nikon, Tokyo, Japan).

#### Apoptosis assay

The effects of diluted solutions of powder extracts on the apoptosis of osteoclast progenitors were analyzed using an Annexin V-FITC/propidium iodide (PI) apoptosis detection kit (403302ES20, Yeasen Biotechnology). According to the manufacturers’ instructions, cells were seeded in 6-well culture plates at a density of 4 × 10^5^ per well. After an incubation period of 24 h, cells in diluted solutions (dilution ratios: 1/4, 1/8, 1/16, 1/32, 1/64 and 1/128) of BG-/Mo-extracts were digested, centrifuged, rinsed, and incubated in 200 µL of PBS containing 5 μL of Annexin V-FITC and 5 μL of PI for 15 min at room temperature. Subsequently, all the obtained cells were rinsed and subjected to flow cytometry (BD Accuri C6, San Jose, CA, USA) to measure the apoptosis rate (%).

### Effects of graded concentrations of Mo-extracts on the differentiation of osteoclast progenitors

To assess the effects of diluted solutions of Mo-extracts on the differentiation of osteoclast progenitors, cells were seeded in 96-well culture plates at a density of 6 × 10^3^ per well. When the cells were attached to the culture plates, the complete medium was changed to diluted (dilution ratio: 1/4, 1/32 or 1/128) BG-/Mo-extract solutions containing 50 ng/mL receptor activator of nuclear factor kappa-В ligand (RANKL; R&D systems, Minneapolis, MN, USA) and 50 ng/mL macrophage colony-stimulating factor (M-CSF; PeproTech, Princeton, NJ, USA); each medium was refreshed every other day. Following a 5-day induction, the cells were subjected to tartrate-resistant acid phosphatase (TRAP) staining. In parallel to TRAP staining, the formation of the F-actin ring and the expression of specific protein markers (MMP9 and NFATc1) of osteoclastogenesis and osteoclastogenesis-related genes (*MMP9*, *NFATc1* and *RANKL*) in osteoclastic cells were evaluated by immunofluorescence staining and qRT–PCR.

### Effects of Mo ions in the culture medium on the differentiation of osteoclast progenitors

Similarly, culture medium containing 3.75 mg·L^−1^ Mo ions (Mo) or a diluted BG-extract solution (dilution ratio: 1/32) containing 3.75 mg·L^−1^ Mo (BG + Mo) was used to assess the influence of the presence of Mo ions in culture medium on the differentiation of osteoclast progenitors; culture medium (Control) and diluted BG-extract solutions (BG-extracts) served as the negative controls, and diluted Mo-extract (Mo-extracts) solutions were used as the positive control. The differentiation of osteoclast progenitors was analyzed via TRAP staining, immunofluorescence staining and qRT–PCR.

#### qRT–PCR

The expression of osteogenesis-related genes (*Runx2*, *ALP* and *SP7*) in BMMSCs and osteoclastogenesis-related genes (*MMP9*, *NFATc1* and *RANKL*) in osteoclastic cells was evaluated by qRT–PCR assay according to previously reported methods.^74,75^ The gene expression levels were normalized using the housekeeping gene *β-actin* (for mRNA), and the primers used in the present study are shown in Table S1.

#### TRAP staining in vitro

Following a 5-day incubation of osteoclast progenitors in graded diluted BG-/Mo-extract solutions containing 50 ng·mL^−1^ of RANKL and M-CSF, the cells were fixed with 10% (v/v) formalin neutral buffer solution for 30 min at room temperature and stained with TRAP solution (TAKARA, Osaka, Japan) for 45 min at 37 °C. Then, the nuclei of the cells were stained with methyl green (TAKARA, Osaka, Japan). Finally, the numbers and the area of TRAP-positive cells with different numbers of nuclei (31–50, 11–30 or 4–10) in each well were quantified from 6 staining samples with Image-Pro Plus software (Media Cybernetics, Bethesda, MD, USA).

#### Immunofluorescence staining in vitro

The specific markers (MMP9 and NFATc1) of osteoclastic cells and the formation of F-actin rings were analyzed by immunofluorescence staining, according to our previously reported methods.^29^ After a 5-day osteoclastic induction, the cells were washed, fixed in 4% paraformaldehyde, and then incubated with osteoclastogenesis-related antibodies, followed by incubation with the corresponding secondary antibodies, Alexa 594 donkey anti-rabbit IgG (1:500, 34206ES60, Yeasen Biotechnology) or Alexa 488 donkey anti-mouse IgG (1:500; 34206ES60, Yeasen Biotechnology), and counterstaining with DAPI (1:500, 40728ES03, Yeasen Biotechnology). The primary antibodies used in the present study were as follows: rabbit anti-MMP9 (1:200; 10375-2-AP, Proteintech) plus TRITC-phalloidin (1:1 000; 40734ES75, Yeasen Biotechnology) and mouse anti-NFATC1 (1:200, 66963-1-Ig, Proteintech) plus FITC-phalloidin (1:1 000; 40735ES75, Yeasen Biotechnology). Confocal images were captured with a Nikon confocal laser microscope (A1 PLUS).

### Effects of Mo ions in the culture medium on mitochondrial biogenesis and ROS production in osteoclasts

Given that the activity of mitochondrial biogenesis and ROS production are closely integrated with osteoclast differentiation,^30,31^ culture medium containing 3.75 mg·L^−1^ Mo ions (3.75) or 10 mg·mL^−1^ Mo ions (10) was used to assess the influence of the presence of Mo ions in culture medium on mitochondrial biogenesis and ROS production in osteoclasts; culture medium without Mo supplementation was set as the control. Mitochondrial biogenesis is mainly orchestrated by peroxisome proliferator–activated receptor-c coactivator 1β (PGC-1β), whose translation is induced by AMP response element–binding protein (CREB) as a result of ROS.^30^ Therefore, PGC-1β and CREB were used to analyze the activity of mitochondrial biogenesis in osteoclasts. Following a 5-day osteoclastic induction, the expression of PGC-1β and p-CREB was analyzed via immunofluorescence staining and Western blotting, while mitochondrial ROS production was analyzed using MitoSOX staining. The primary antibodies used in the Western blot assay were as follows: antibodies targeting PGC-1β (1:100; sc-373771, Santa Cruz Biotechnology, CA, USA), p-CREB (1:1 000; bs-0036R, Bioss, Beijing, China), CREB (1:1 000;12208-1-AP, Proteintech) and β-actin (1:5 000; P20536-1-AP, Proteintech). The immunofluorescence staining was performed as described in Section 2.8.3. The following primary antibodies were used in the present study: rabbit anti-p-CREB (1:200; bs-0036R, bioss) plus mouse anti-PGC-1β (1:100; sc-373771, Santa Cruz Biotechnology). The mitochondrial ROS levels in osteoclasts were determined using a MitoSOX probe (Invitrogen, Carlsbad, CA, USA). Cells were rinsed and immersed in culture medium supplemented with 5 μM MitoSOX at 37 °C for 30 min in the dark. Subsequently, the incubated cells were washed gently with PBS, and immunofluorescent images were captured using a Nikon confocal laser microscope (A1 PLUS).

### Transplantation of scaffolds into canines with critical-size alveolar bone defects

To investigate how the Mo-scaffold affected in vivo osteogenesis/osteoclastogenesis responses and ultimate bone regeneration outcomes, artificial critical-size alveolar bone defects in 12 male beagle dogs (18–24 months, weighing 10–12 kg) were used for transplantation experiments in the present study. All animal procedures were conducted in accordance with international ethical guidelines and were approved by the Laboratory Animal Care and Welfare Committee, School of Stomatology, Fourth Military Medical University. Similar to the methods we reported previously,^29^ the mandibular first and third premolars in both jaw quadrants were extracted to prepare space for further defect creation. Following an 8-week wound healing period, 4 × 5 cm (width × height) one-wall intrabony defects were carefully created at the distal site of the second and at the mesial site of the fourth mandibular premolar teeth, and each defect was randomized for placement of defect size-matched 3D-printed BG- or Mo-scaffolds. All experimental canines were fed a canned soft dog food diet for 7 consecutive days post-surgery before resuming routine feeding and animal care.

### Bone tissue regeneration and in vivo osteogenic/osteoclastogenic responses

#### Bone tissue regeneration following Mo-scaffold transplantation

Alveolar bone regeneration in vivo was evaluated via radiological analysis, micro-CT analysis and methylene blue-acid fuchsin/hematoxylin-eosin (H&E) staining.^29^ X-ray radiography was used to track the remaining BG-/Mo-scaffold transplants and evaluate the dynamic changes in alveolar bone defects at 0, 1, 2, 4 and 8 weeks post-surgery. At 1 and 8 weeks post-surgery, the lower jaw between the second and fourth premolars in both quadrants was collected and analyzed via micro-CT scanning. The newly regenerated alveolar bone was evaluated in terms of morphometric bone parameters, including bone volume/tissue volume (BV/TV), trabecular separation (Th.Sp), trabecular number (Tb.N), and trabecular thickness (Tb.Th) using the commercial software VGStudioMAX 3.0 (Volume Graphics). Specimens from 8 weeks post-surgery were also subjected to methylene blue-acid fuchsin staining and H&E staining to evaluate the newly formed bone tissues and blood vessels across bone defects.

#### Local osteogenic/osteoclastogenic responses during the wound healing cascade

The effects of scaffold transplants on in vivo osteogenesis were evaluated via alkaline phosphatase (ALP) staining at 1, 2 and 4 weeks post-surgery. The hard tissue sections were stained with ALP substrate solution (TAKARA, Osaka, Japan) for 3 h at 37 °C. Then, microscopic images of ALP-stained samples were captured by stereomicroscopy (Leica) after washing twice with double-distilled water. The number of ALP-positive cells in 3 randomly selected views per specimen was calculated using Image-Pro Plus 6.0 software (Media Cybernetics). In parallel to osteogenic responses, the effects of scaffold transplants on osteoclastogenesis in vivo were evaluated by TRAP staining. The hard tissue sections were stained with TRAP solution (TAKARA, Osaka, Japan) for 2 h at 37 °C. Then, microscopic images were captured under a stereomicroscope (Leica).

### Statistical analysis

GraphPad Prism software (version 8.0, San Diego, CA, USA) was used to perform statistical analysis was performed using. Data were collected from at least three cell lines and are expressed as the means ± standard deviations (SDs). The exact sample size for each experimental group is shown in a dot plot in the figures and indicated in the figure legends. The significance of differences between two groups were analyzed by the Student’s *t* test; comparisons among multiple groups were analyzed by one-way or two-way analysis of variance (ANOVA) followed by Tukey’s multiple-comparison post hoc test. Statistical significance was noted as *P < 0.05, **P < 0.01 or ***P < 0.001.

## Supplementary information

Supporting information

## Data Availability

All data needed to evaluate the conclusions in the paper are present in the paper and additional data related to this paper may be requested from the authors.
