# Supplementary material for: A 3D-printed molybdenum-containing scaffold exerts dual pro-osteogenic and anti-osteoclastogenic effects to facilitate alveolar bone repair
Source: Int J Oral Sci. 2022 Sep 5;14:45. doi: 10.1038/s41368-022-00195-z (PMC9445063; doi:10.1038/s41368-022-00195-z)
Supplement: Supplementary file 1 — Supporting information [file 41368_2022_195_MOESM1_ESM.pdf]

Supporting Information

A 3D-printed molybdenum-containing scaffold exerts dual pro-osteogenic and anti-osteoclastogenic effects to facilitate alveolar bone repair

Running title: A molybdenum-containing scaffold for bone repair

Bei-Min Tian,[1] Xuan Li,[1] Jiu-Jiu Zhang,[1] Meng Zhang,[2] Dian Gan,[1] Dao-Kun Deng,[1] Li-Juan Sun,[1] Xiao-Tao He,[1] Chengtie Wu,[2*] and Fa-Ming Chen[1*]

[1]Department of Periodontology, State Key Laboratory of Military Stomatology and National Clinical Research Center for Oral Diseases, Shaanxi Engineering Research Center for Dental Materials and Advanced Manufacture, School of Stomatology, Fourth Military Medical University, Xi'an, P. R. China

[2]State Key Laboratory of High-Performance Ceramics and Superfine Microstructure, Shanghai Institute of Ceramics, Chinese Academy of Sciences, Shanghai, P. R. China

[*]**Corresponding author**

Chengtie Wu, Email: chengtiewu@mail.sic.ac.cn or Fa-Ming Chen, Email: cfmsunhh@fmmu.edu.cn

These authors contributed equally: Bei-Min Tian and Xuan Li

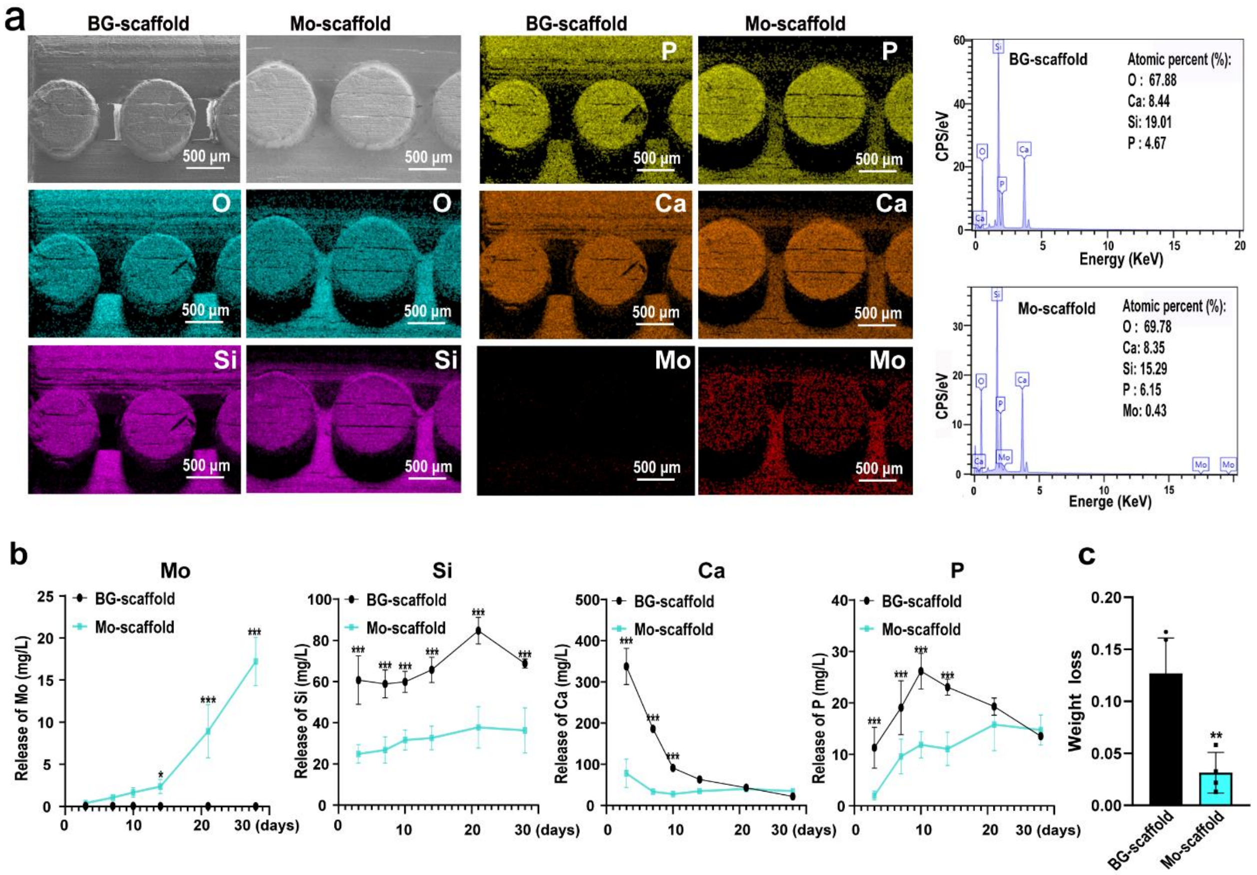

Fig. S1. The presence of specific elements and the degradation behavior of the 3D-printed bioactive glass ceramic scaffolds with or without molybdenum (BG- or Mo-scaffolds). (a) Energy dispersive spectroscopy (EDS) mapping and ion analysis showing that O, Si, P, and Ca were present in both scaffolds, but only Mo was present in the Mo scaffold. (b) Cumulative ion release profiles of Mo, Si, Ca or P ions from BG- and Mo-scaffolds immersed in Tris-HCl solution ($n = 5$). (c) Weight loss of BG- and Mo-scaffolds immersed in Tris-HCl solution for 28 days ($n = 4$). The data are shown as the mean ± SD; *$p < 0.05$, **$p < 0.01$ and ***$p < 0.001$ indicate significant differences between BG- and Mo-scaffolds at the same testing time point or between the indicated columns.

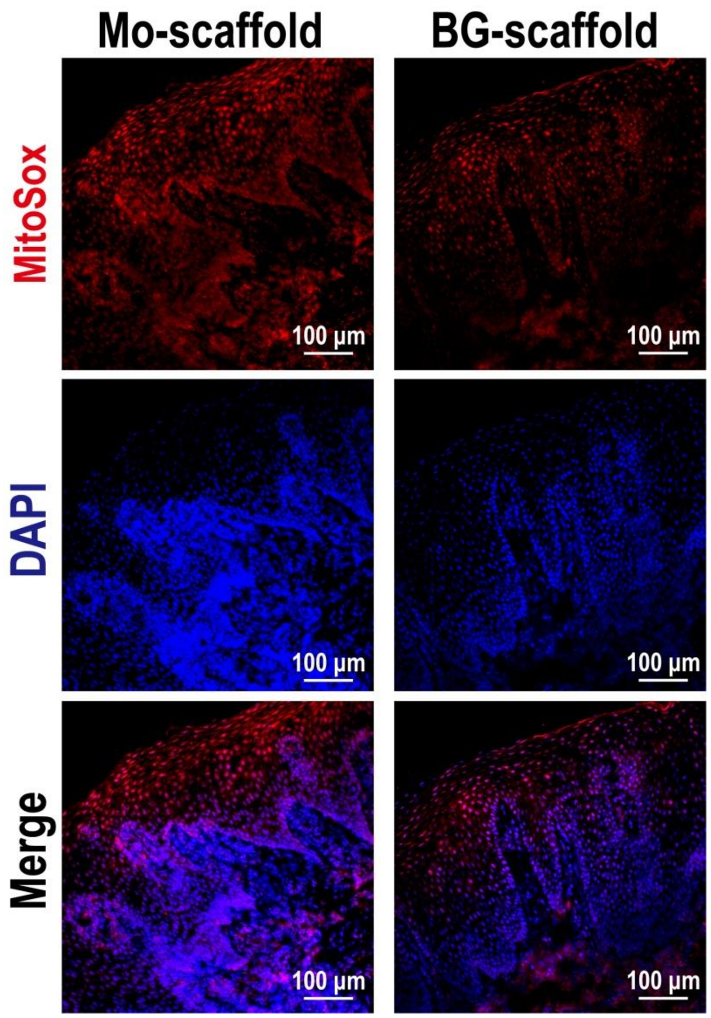

Fig. S2. Representative confocal images showing the mitochondrial ROS levels in gingiva covering BG-scaffolds or Mo-scaffolds at 2 weeks post-surgery (MitoSOX staining). Scale bar = 20 μm.

Table S1. qRT–PCR primer sequences used in the present study.

Gene	Full name	Primers	Sequences (5'-3')
Runx2	*Runt-related transcription factor-2*	Forward	GACTGTGGTTACCGTCATGGC
		Reverse	ACTTGGTTTTTCATAACAGCGGA
ALP	*Alkaline phosphatase*	Forward	CTTCTTGCTGGTGGAAGGA
		Reverse	AAAACGTGGGAATGATCAGC
SP7	*SP7 transcription factor-2*	Forward	GGGGAAAGGAGGCACAAAG
		Reverse	GTGAGGGAAGGGTGGGTAGTC
NFATc1	*Nuclear factor of activated T cells 1*	Forward	GCCTCGAACCCTATCGAGTG
		Reverse	TCCCGGTCAGTCTTTGCTTC
MMP-9	*Matrix metalloprotein-9*	Forward	*CGCTCATGTACCCGCTGTAT*
		Reverse	*CCGTGGGAGGTATAGTGGGA*
RANKL	*Receptor Activator of Nuclear Factor Ligand*	Forward	*CCGAGACTACGGCAAGTACC*
		Reverse	CTGCGCTCGAAAGTACAGGA

Table S2. Concentrations of Mo, Si, Ca and P ions in graded dilute BG- and Mo-extracted solutions. #

Dilution ratio / Element	1/4		1/32		1/128	
	BG-extract	Mo-extract	BG-extract	Mo-extract	BG-extract	Mo-extract
Mo (mg/L)	0.00 ± 0.00	31.65 ± 0.57***	0.01 ± 0.01	3.75 ± 0.33***	0.00 ± 0.00	1.09 ± 0.08***
Si (mg/L)	34.60 ± 0.96	23.08 ± 0.59***	4.33 ± 0.25	2.89 ± 0.16***	1.09 ± 0.09	0.73 ± 0.03***
Ca (mg/L)	57.20 ± 3.31	80.50 ± 1.40***	58.96 ± 4.48	59.13 ± 5.12	55.95 ± 4.37	59.58 ± 2.52
P (mg/L)	25.60 ± 0.01	26.70 ± 0.14**	32.78 ± 2.34	33.25 ± 2.66	32.28 ± 2.26	34.50 ± 1.47

The data are shown as the mean ± SD ($n = 4$); **$p < 0.01$ and ***$p < 0.001$ indicate significant differences between BG extracts and Mo extracts at the same dilution ratio.
